# Exposure to neighborhood concentrated poverty is associated with faster decline in episodic memory among midlife women

**DOI:** 10.1002/alz.70139

**Published:** 2025-04-06

**Authors:** Jinshil Hyun, Mary Schiff, Charles B. Hall, Bradley M. Appelhans, Emma Barinas‐Mitchell, Rebecca C. Thurston, Carrie A. Karvonen‐Gutierrez, Monique M. Hedderson, Imke Janssen, Carol A. Derby

**Affiliations:** ^1^ Saul R. Korey Department of Neurology Albert Einstein College of Medicine Bronx New York USA; ^2^ UPMC Children's Hospital of Pittsburgh Pittsburgh Pennsylvania USA; ^3^ Department of Epidemiology & Population Health Albert Einstein College of Medicine Bronx New York USA; ^4^ Department of Family and Preventive Medicine Rush University Medical Center Chicago Illinois USA; ^5^ Department of Epidemiology University of Pittsburgh Pittsburgh Pennsylvania USA; ^6^ Department of Psychiatry University of Pittsburgh Pittsburgh Pennsylvania USA; ^7^ Department of Epidemiology University of Michigan Ann Arbor Michigan USA; ^8^ Division of Research Kaiser Permanente Northern California Oakland California USA

**Keywords:** cognitive decline, delayed recall, episodic memory, hot spots analysis, immediate recall, longitudinal analysis, midlife, neighborhood concentrated poverty, race, socioeconomic

## Abstract

**INTRODUCTION:**

Neighborhood‐level socioeconomic status (nSES) is associated with risk for cognitive impairment, but prior studies assessed nSES within an individual's own residential area without considering the distribution of nSES among adjacent areas.

**METHODS:**

Using up to 14 years of data from the Study of Women's Health Across the Nation (*N* = 1391, mean age = 54), we examined whether geographic clustering of concentrated neighborhood poverty was associated with cognitive decline over midlife.

**RESULTS:**

Greater neighborhood concentrated poverty was associated with faster decline in episodic memory but not in processing speed or working memory. Living in high concentrated poverty areas was linked to a 7% episodic memory decline per decade (both immediate and delayed recall), with Black women experiencing the steepest decline at 10% per decade (delayed recall).

**DISCUSSION:**

Women living in concentrated poverty areas exhibited accelerated decline in episodic memory during midlife. Neighborhood concentrated poverty may impact risk for future cognitive impairment and ADRD.

**Highlights:**

- Living in concentrated poverty areas predicted a more rapid episodic memory decline.
- This pattern was most pronounced among Black women.
- The cohort was a racially/ethnically diverse cohort of midlife women across the US.
- Neighborhood concentrated poverty may contribute to the risk of ADRD.

## 1 BACKGROUND

Geographic inequality between richer and poorer areas has grown over the past decades.^1^

Prior studies have documented that living in a socioeconomically deprived neighborhood contributes to disparities in various health outcomes including cardiovascular risks, chronic conditions, mental health, self‐rated health, and mortality.^2^, ^3^ The ecological framework provides theoretical explanations, by positing that features of social and built environment exist above and before the individual, and that the environment constrains, induces, and shapes individuals' health‐related behavior and health outcomes.^4^ For example, socio‐economically deprived neighborhoods are characterized by less optimal physical/built (e.g., amenities, green space) and social (e.g., social cohesion, safety) contexts, and may have downstream effects on health‐related behaviors (e.g., physical inactivity, less social engagement, less cognitive stimulation), which in turn may affect individuals’ health outcomes.^5^, ^6^ In line with this conceptual framework, previous studies have also documented evidence for cognitive health outcomes, such that greater neighborhood deprivation is associated with lower levels of cognitive performance, with faster rates of cognitive decline, and with more rapid development of Alzheimer's disease neuropathology and cortical changes.^7^, ^8^, ^9^, ^10^, ^11^, ^12^, ^13^, ^14^, ^15^, ^16^

However, prior evidence in the association between neighborhood socioeconomic status (nSES) and cognitive health outcomes have some limitations. First, most studies have assessed neighborhood deprivation within specific administrative boundaries (e.g., census tract) without considering the distribution of SES among adjacent neighborhood areas (i.e., spatial segregation).^8^, ^9^, ^11^, ^12^, ^13^, ^17^, ^18^, ^19^, ^20^, ^21^, ^22^ However, people spend substantial amounts of their days in areas beyond their residential locations.^23^ Individuals who live in a low nSES community that is surrounded by areas with similarly low nSES will have poorer access to health‐related neighborhood resources such as healthy grocery stores, safe greenspace, or medical facilities, in their day‐to‐day lives. Second, most prior studies, with a few exceptions,^11^, ^18^ have utilized neighborhood exposure measures assessed at a single time point.^8^, ^12^, ^13^, ^21^, ^22^ Given that people can move and neighborhoods themselves may change over time^24^ (e.g., gentrification), this methodological approach may induce exposure misclassification, thereby biasing observed associations. Third, most studies have used global or composite cognitive scores, such as Mini‐Mental State Examination (MMSE) or its adapted versions,^11^, ^19^, ^20^, ^21^ which have low sensitivity for detecting cognitive impairment.^25^ Further, as a global measure, they provide little ability to understand any associations of nSES with specific cognitive domains. Only a few studies have examined effects of neighborhood deprivation on long‐term changes in specific cognitive domains such as processing speed and memory, and results have been inconsistent.^8^, ^20^

The overall aim of this study was to examine how living in an area of high concentrated poverty (e.g., a high poverty neighborhood area surrounded by similarly high poverty neighborhoods [i.e., hot spots]) was associated with longitudinal changes in several cognitive domains among midlife women. To overcome limitations of prior studies, (i) we considered both the area of individual residence and adjacent neighborhood areas to define nSES, (ii) we considered longitudinal address information to capture cumulative effects from changes in residence over time, and (iii) we included longitudinal measures of the cognitive function domains of processing speed, working memory, and verbal episodic memory. Given that midlife is a critical stage in the life‐course to implement interventions and promote health behaviors to prevent future disease and impairment,^26^, ^27^ it is critical to identify which upstream neighborhood‐level factors shape midlife cognitive health outcomes. We hypothesized that exposure to higher levels of neighborhood concentrated poverty throughout midlife would be associated with more rapid declines in cognitive performance. For an exploratory aim, we also examined whether associations between concentrated poverty and cognitive trajectories would vary across racial/ethnic groups.

## 2 METHODS

### 2.1 Participants

The current analysis used data from the Study of Women's Health Across the Nation (SWAN), a longitudinal cohort study of women's health from midlife into older adulthood.^28^ Eligibility at study baseline (in 1996) included ages between 42 and 52 years, having an intact uterus and at least one intact ovary, not being pregnant or breast feeding, having menstrual bleeding, and no prior use of exogenous hormones affecting ovarian or pituitary function within the past 3 months. Five racial or ethnic groups were recruited across seven US study sites: Black (Boston, MA, USA; Chicago, IL, USA; Detroit, MI, USA; Pittsburgh, PA, USA), Chinese (Oakland, CA, USA), Hispanic (Newark, NJ, USA), Japanese (Los Angeles, CA, USA), and non‐Hispanic White (all sites).

The present study includes data collected through the 15th follow‐up visit (2015–2017). Cognitive testing started at SWAN Visit 4 and was repeated at Visits 6, 7, 8/9, 10, 12, 13, and 15. Prior SWAN studies have shown improved cognitive performance across the initial three assessments, suggesting that this performance gain primarily reflected learning effects.^29^, ^30^ To minimize the influence of learning effects in the longitudinal analysis, the cognitive baseline for the present analysis was set at the third cognitive assessment (Visit 7), where learning effects were no longer apparent.^31^ Consequently, analyses of current study included women who completed their third cognitive assessment at Visit 7.

The analytic sample included women who had residential address data and completed their third cognitive assessment at Visit 7. Data from the Newark, NJ and Boston, MA sites were not included in the analytic sample because the third cognitive assessment did not occur at Visit 7 at the NJ site and the Boston SWAN site did not contribute residential address data. The initial sample size from five US study sites (Chicago, IL, USA; Detroit, MI, USA; Pittsburgh, PA, USA; Oakland, CA, USA; and Los Angeles, CA, USA) was 2336. Among these participants, 856 women whose cognitive baseline was not Visit 7 were excluded. Additionally, we excluded women having history of stroke (*N* = 14), myocardial infarction (*N* = 18), or angina (*N* = 27) prior to the cognitive baseline, women with missing education variable (*N* = 9), women who did not complete cognitive assessments in the same language at each visit (*N* = 18), and those with missing neighborhood concentrated poverty measures (*N* = 3). Study visits were censored for incident stroke during follow‐up (*N* = 40); the final analytic sample included 1391 women across 7199 longitudinal visits (Figure S1). All participants provided written informed consent at each study visit, and approval was obtained from Institutional Review Boards at each SWAN clinical site.

**RESEARCH IN CONTEXT**

1. **Systematic review**: Previous studies have documented evidence that living in lower levels of neighborhood‐level socioeconomic status (nSES) areas is associated with cognitive impairment and risks for ADRD. These studies typically assessed nSES within specific administrative boundaries within individuals’ own residential areas, without accounting for the distribution of nSES among adjacent areas.
2. **Interpretation**: Living in an area of high concentrated poverty (e.g., a high poverty neighborhood area surrounded by similarly high poverty neighborhoods) was associated with faster rates of decline in episodic memory throughout midlife; this pattern was most pronounced among Black women. Neighborhood concentrated poverty may contribute to the risk of ADRD beyond normative cognitive aging processes.
3. **Future directions**: Future studies need to identify psychosocial and biological mechanisms underlying this association. Policy and environmental interventions would be required to mitigate cognitive health disparities in economically segregated areas, especially among minoritized people as early as midlife.

### 2.2 Neighborhood concentrated poverty

Residential address histories were maintained from baseline through follow‐up visit 11 (2007–2009). All addresses were geocoded to the census tract level based on the 2010 census boundary classifications. Neighborhood concentrated poverty level was calculated using the local Getis‐Ord (Gi^*^) spatial statistic based on poverty data from the US census. Census tract‐level poverty data between 1990 and 2010 were obtained from the Longitudinal Tract Database (Brown University), which normalized estimates from different decennial census years to the 2010 boundary classifications; this allowed longitudinal comparison across the same geographic boundaries across time).^32^, ^33^ We obtained absolute poverty rates – defined as the proportion of residents living 150% below the federal poverty limit – for each census tract located within each local SWAN site area, and linearly interpolated estimates for years occurring between the decennial censuses. We then used the local Gi^*^ spatial statistic,^34^, ^35^ a spatially weighted measure that evaluates the extent to which the poverty rate of the focal tract and its neighboring tracts deviate from the mean poverty rate of all tracts located within the set of counties comprising each local SWAN site area. Gi^*^ Z‐scores for census tracts were calculated annually within each local SWAN site area separately, using a first‐order queen contiguity spatial weight matrix. A significantly positive z‐score indicates that a given focal tract with a high poverty rate is surrounded by neighboring tracts with similarly high poverty rates, and a significantly negative z‐score indicates that a given focal tract with a low poverty rate is surrounded by neighboring tracts with similarly low poverty rates. A z‐score around zero indicates no geographic clustering by poverty rates. The Gi^*^ scores were linked with each participant's geocoded census tract of residence at each visit‐year so that we could take relocation into account. To estimate how cumulative effect of neighborhood concentrated poverty exposure (Visit 0 to Visit 7) predicts changes in future cognitive performance (Visit 7 to Visit 15), longitudinal Gi^*^ scores were summarized as a time‐invariant variable by averaging Gi^*^ scores between baseline visit (Visit 0) and cognitive baseline (Visit 7). The final, time‐invariant neighborhood concentrated poverty variable was categorized into three levels by 95% confidence level: low concentrated poverty (Gi^*^ z‐scores < −1.96; cold spots), moderate concentrated poverty (Gi^*^ −1.96 to 1.96), and high concentrated poverty (Gi^*^ > 1.96; hot spots). The Gi^*^ Z‐scores below −1.96 or above +1.96 suggest that there is less than a 5% probability that this clustering is due to random chance. The Gi^*^ scores between −1.96 and +1.96 indicate that there is no significant spatial clustering in these areas. We also used continuous, time‐invariant Gi^*^ Z‐scores in sensitivity analyses. See Schiff and colleagues^36^ for details.

### 2.3 Cognitive assessments

Various cognitive domains were assessed including processing speed, working memory, and verbal episodic memory. Processing speed was assessed with the symbol digit modalities test, in which participants matched numbers to symbols. The test score was the number of correct matches (range 0–110) in 90 s.^37^ Working memory, the ability to manipulate information held in memory, was assessed by the digit span backwards test (DSB).^38^ Participants are read strings of single‐digit numbers and asked to repeat them backwards. Number strings ranged from 2 to 7 digits in length, with two trials at each length. The test was stopped after completing all trials or when the response was incorrect for both trials of a given length. The test was scored as the number of correct trials (range 0–12).^38^ Verbal episodic memory was evaluated using the East Boston Memory test (EBMT) immediate and delayed recall. Respondents are read a brief paragraph and asked to recall story elements immediately and after a 10‐min delay. The total score is the number of correct elements recalled (range 0–12).^39^ For each cognitive test, higher scores reflect better cognitive performance.

### 2.4 Covariates

Prior literature has listed demographic (race/ethnicity, education, financial situation), behavioral (alcohol consumption, smoking), and menopause‐related factors that may be associated with cognitive trajectories.^29^, ^40^, ^41^ These factors may confound the associations between neighborhood poverty and cognition and were included as covariates in the main analyses. Time‐invariant covariates included study site, race or ethnicity, and education (greater than high school diploma vs. high school or less). Time‐varying covariates included self‐reported alcohol use (none/infrequent vs. twice a week or more), current smoking, self‐reported financial strain (not very hard vs. somewhat/very hard to pay for basic necessities), oral contraceptive or reproductive hormone use, and menopause status [(i) pre‐menopause or early peri‐menopause (at least one menstrual period within the past 3 months), (ii) late peri‐menopause (3 consecutive months of amenorrhea), (iii) natural post menopause (≥12 months of amenorrhea), and (iv) post menopause by bilateral oophorectomy or hysterectomy].

Based on the prior literature highlighting the potential mediating roles that physical activity and cardiovascular risk factors can play in the associations between neighborhood poverty and cognition,^5^, ^6^, ^42^ physical activity and Atherosclerotic Cardiovascular Disease (ASCVD) risk score were included as potential mediators in sensitivity analyses. The physical activity measure is based upon the total score from the Kaiser Physical Activity Survey.^43^ As physical activity scores were not available at all visits, we used the physical activity closest to the cognitive baseline (Visit 6). The ASCVD score is a measure of 10‐year risk of developing a first atherosclerotic cardiovascular disease event based on race specific risk scores including use of blood pressure medication, current smoking status, current diabetes status, log‐transformed values of age, total cholesterol, HDL cholesterol, and systolic blood pressure, as developed by a working group of the American Heart Association and the American College of Cardiology using the pooled cohort data from NHLBI‐sponsored longitudinal studies.^44^ For the present analyses, ASCVD score at the cognitive baseline (Visit 7) were used.

### 2.5 Statistical analysis

Descriptive characteristics at the cognitive baseline (SWAN Visit 7) were compared for individuals from different concentrated poverty areas. Categorical variables were compared with the *χ* ^2^ tests; when the frequencies in any cell were below 5, Fisher's exact tests with Monte Carlo simulation were conducted instead. Continuous variables were compared using ANOVA when the outcome is normally distributed and using Kruskal–Wallis tests when the outcome is not normally distributed.

Mixed effects models were used to account for the nested structure of the data (i.e., visits within persons) using a random intercept for person and a random slope for time. Because the median number of participants per census tract in any given visit year was just one to two women, census‐tract‐level random effect was not incorporated.

To test our main aim (i.e., effects of neighborhood concentrated poverty on cognition), baseline levels and rates of change in each cognitive test were modeled as a function of neighborhood concentrated poverty, within‐person time index (time from cognitive baseline, in years), time‐invariant covariates (baseline age, education, race or ethnicity, study site), and time‐varying covariates (smoking, alcohol use, menopausal status, financial strain). Processing speed and working memory scores were modeled with linear mixed‐effects regression (SAS PROC MIXED). Verbal episodic memory (both immediate and delayed recall) scores were modeled using a Tobit model (SAS PROC QLIM) with random effects, which allowed to estimate linear relationships between variables when there was a ceiling effect in the dependent variable. To test our second aim (i.e., racial differences in the effects of neighborhood concentrated poverty and cognition), we included interaction terms of neighborhood × race and neighborhood × time × race in the main analyses. All analyses were performed using SAS 9.4 (SAS Institute).

Several sensitivity analyses were conducted. First, we replicated findings by using Gi^*^ scores with a second‐order spatial weight matrix instead of a first‐order matrix to weight the values of neighboring tracks. Although a first‐order queen contiguity matrix includes immediate neighbors that share common points with a focal tract, a second‐order queen matrix would consider the broader neighbors of the immediate neighbors as well. Second, to examine whether individual factors such as physical activity and cardiovascular disease risk factors accounted for the associations between neighborhood measures and cognition, we adjusted for baseline physical activity and ASCVD.

## 3 RESULTS

### 3.1 Descriptive statistics

**FIGURE 1:**
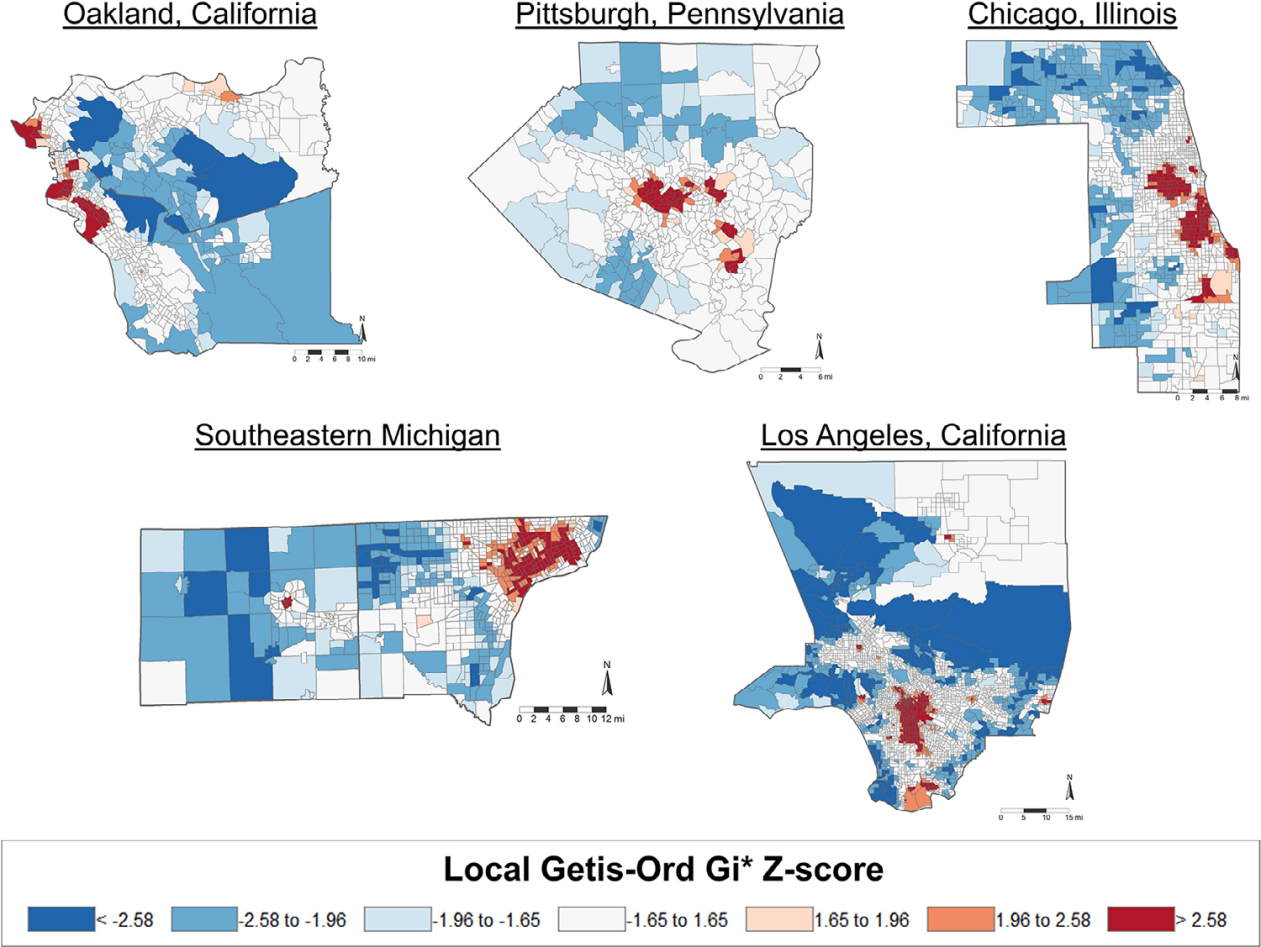
Spatial distribution of high concentrated poverty (i.e., red areas) and low concentrated poverty (i.e., blue areas) located throughout each SWAN site in 2003.

Descriptive statistics for the analytic sample (*N* = 1391) are shown in Table 1. At cognitive baseline, the mean age was 53.6 years (range = 49 to 60), and 54.1% of women were natural post‐menopausal. Approximately 24% of our sample were Black, 49% were White, 12% were Chinese, and 15% were Japanese. Eighteen percent had high school education or less, and financial strain was reported by 23%. Mean follow‐up from cognitive baseline was 10 years (range = 0 to 13.5 years), and the mean number of longitudinal assessments was 5.2 (range = 1–6). Five‐point‐five percent of the sample lived in high concentrated poverty neighborhoods, 71.4% in moderate concentrated poverty neighborhoods, and 23.1% lived in low concentrated poverty areas. Women living in higher levels of concentrated poverty neighborhoods drank alcohol less frequently, were less likely to have post menopause by bilateral oophorectomy or hysterectomy, had lower levels of education, reported greater financial strain, were more likely to be Black or Chinese, were less engaged in physical activity, and had greater ASCVD risk score. Length of follow‐up time did not differ by neighborhood concentrated poverty, indicating that differential attrition by neighborhood group is less likely. Figure 1 displays the spatial distribution of high concentrated poverty areas and low concentrated poverty areas located throughout each SWAN site region.

**TABLE 1:** Descriptive statistics at cognitive baseline (% (Sample size) or Mean [SD]).

Variable	All (*N* = 1391)	Low concentrated poverty (*N* = 321)	Moderate concentrated poverty (*N* = 993)	High concentrated poverty (*N* = 77)	*p*‐value
Age	53.6 (2.7)	53.6 (2.7)	53.6 (2.7)	53.2 (2.3)	0.493
Current smoker	10.2% (141)	5.9% (19)	11.8% (116)	7.8% (6)	0.009
Alcohol use: Frequent drinking (2+/week)	21.5% (296)	34.1% (109)	17.8% (174)	17.1% (13)	<0.001
Menopause status					
Post by BSO/hysterectomy	7.1% (93)	6.1% (18)	8.1% (75)	0% (0)	0.019
Natural post	54.1% (704)	57.6% (170)	52.6% (490)	58.7% (44)	
Late peri	10.4% (135)	7.1% (21)	11.1% (103)	14.7% (11)	
Early peri/pre	28.4% (370)	29.2% (86)	28.3% (264)	26.7% (20)	
High school or less	18% (250)	9.7% (31)	19.6% (195)	31.2% (24)	<0.001
Financial strain	23.4% (320)	17.6% (56)	24.7% (241)	31.5% (23)	0.009
Race/Ethnicity					
Non‐Hispanic White	48.8% (679)	64.8% (208)	44.8% (445)	33.8% (26)	<0.001
Black	23.7% (330)	4.4% (14)	28.4% (282)	44.2% (34)	
Chinese	12.1% (168)	3.7% (12)	14% (139)	22.1% (17)	
Japanese	15.4% (214)	27.1% (87)	12.8% (127)	0% (0)	
Study site					
Michigan	18.1% (252)	4.1% (13)	24.1% (239)	0% (0)	<0.001
Chicago	15.5% (215)	19.3% (62)	15.4% (153)	0% (0)	
Oakland	21.9% (304)	7.8% (25)	25.1% (249)	39% (30)	
Los Angeles	26.2% (364)	63.6% (204)	16% (159)	1.3% (1)	
Pittsburgh	18.4% (256)	5.3% (17)	19.4% (193)	59.7% (46)	
Physical activity	7.60 (1.72)	7.87 (1.66)	7.52 (1.74)	7.48 (1.71)	0.006
Atherosclerotic cardiovascular disease (ASCVD) risk score	0.03 (0.04)	0.02 (0.02)	0.03 (0.05)	0.03 (0.05)	<0.001
Number of longitudinal visits since cognitive baseline	5.2 (1.3)	5.2 (1.3)	5.2 (1.3)	5.2 (1.4)	0.973
Years of follow‐up since cognitive baseline	10.2 (3.5)	10.2 (3.4)	10.2 (3.4)	10.5 (3.6)	0.719
***Cognition***					
Processing speed	59.0 (10.1)	62.4 (9.1)	58.2 (10.1)	55.4 (10.9)	<0.001
Working memory	6.9 (2.3)	7.4 (2.2)	6.8 (2.3)	6.1 (1.7)	<0.001
Immediate recall	10.4 (1.6)	10.7 (1.4)	10.3 (1.7)	10.5 (1.5)	<0.001
Delayed recall	10.3 (1.7)	10.5 (1.5)	10.2 (1.8)	10.4 (1.7)	0.006

*Note*: Chi‐square tests were conducted for the comparisons of smoking, alcohol use, education, and financial strain. Fisher's exact tests were conducted for menopause status, race/ethnicity, and study site. Kruskal‐Wallis test was conducted for ASCVD risk score comparison due to the skewed distribution. ANOVA was conducted for other continuous variables.

### 3.2 Effects of neighborhood concentrated poverty on cognition

First, we examined the effects of neighborhood concentrated poverty on cognitive trajectories (Tables 2, 3, 4, 5). We calculated coefficients and confidence intervals (CIs) for each level of concentrated poverty (e.g., high, intermediate, low) from results of linear mixed models (processing speed, working memory) and Tobit models (immediate and delayed recall) relating neighborhood concentrated poverty on levels and rates of change in cognitive function. Consequently, the estimates presented in Tables 2, 3, 4, 5 represent the coefficients for each level of neighborhood concentrated poverty, rather than differences relative to a reference group.

**TABLE 2:** Estimated levels and changes in processing speed by neighborhood concentrated poverty.

Neighborhood concentrated poverty	Estimate	SE	95% CI	
			Lower	Upper
Low^a^	64.124	0.759	62.635	65.612
Moderate	63.299	0.686	61.954	64.645
High^a^	61.406	1.415	58.631	64.180
Low × Time	−0.298	0.031	−0.359	−0.236
Moderate × Time	−0.307	0.021	−0.348	−0.266
High × Time	−0.246	0.065	−0.373	−0.119

*Note*: Covariates were baseline age, education, race, study site (time‐invariant), pay for basics, smoking, alcohol use, menopausal status, and hormone use (time‐varying).

^a^ There were significant differences in levels in processing speed between low and high concentrated poverty. There were no significant differences in rates of change among low, moderate, and high concentrated poverty.

**TABLE 3:** Estimated levels and changes in working memory by neighborhood concentrated poverty.

Neighborhood concentrated poverty	Estimate	SE	95% CI	
			Lower	Upper
Low^a^	8.415	0.194	8.034	8.795
Moderate	8.184	0.171	7.849	8.519
High^a^	7.840	0.280	7.292	8.389
Low × Time	−0.008	0.010	−0.028	0.012
Moderate × Time	−0.021	0.006	−0.032	−0.010
High × Time	−0.017	0.016	−0.048	0.013

*Note*: Covariates were baseline age, education, race, study site (time‐invariant), pay for basics, smoking, alcohol use, menopausal status, and hormone use (time‐varying).

^a^ Levels of working memory were significantly different between low and high concentrated poverty. There were no significant differences in rates of change among low, moderate, and high concentrated poverty.

**TABLE 4:** Estimated levels and changes in episodic memory—immediate recall by neighborhood concentrated poverty.

Neighborhood concentrated poverty	Estimate	SE	95% CI	
			Lower	Upper
Low^a^	11.268	0.164	10.946	11.590
Moderate^a^	11.337	0.214	10.918	11.755
High^a^	11.913	0.314	11.297	12.529
Low × Time^b^	0.005	0.014	−0.022	0.033
Moderate × Time^b^	−0.028	0.008	−0.044	−0.011
High × Time^b^	−0.085	0.027	−0.138	−0.032

*Note*: Covariates were baseline age, education, race, study site (time‐invariant), pay for basics, smoking, alcohol use, menopausal status, and hormone use (time‐varying).

^a^ Levels of immediate memory were significantly different between low and high, and between moderate and high concentrated poverty.

^b^ Rates of change in immediate memory were significantly different among all levels of concentrated poverty.

**TABLE 5:** Estimated levels and changes in episodic memory—delayed recall by neighborhood concentrated poverty.

Neighborhood concentrated poverty	Estimate	SE	95% CI	
			Lower	Upper
Low^a^	10.937	0.164	10.617	11.258
Moderate^a^	11.125	0.213	10.708	11.542
High^a^	11.612	0.312	11.001	12.222
Low × Time^b^	0.013	0.014	−0.014	0.040
Moderate × Time^b^	−0.037	0.008	−0.053	−0.021
High × Time^b^	−0.081	0.026	−0.132	−0.029

*Note*: Covariates were baseline age, education, race, study site (time‐invariant), pay for basics, smoking, alcohol use, menopausal status, and hormone use (time‐varying).

^a^ Levels of delayed recall were significantly different between low and high, and between moderate and high concentrated poverty.

^b^ Rates of change in delayed recall were significantly different between low and high, and between low and moderate concentrated poverty.

For each cognitive domain, estimates for the main effect of neighborhood concentrated poverty (high, moderate, low) indicate mean cognitive performance at cognitive baseline. Estimates for the interaction between neighborhood concentrated poverty and time indicate rates of change in cognitive performance; negative values indicate more accelerated cognitive decline over time.

**FIGURE 2:**
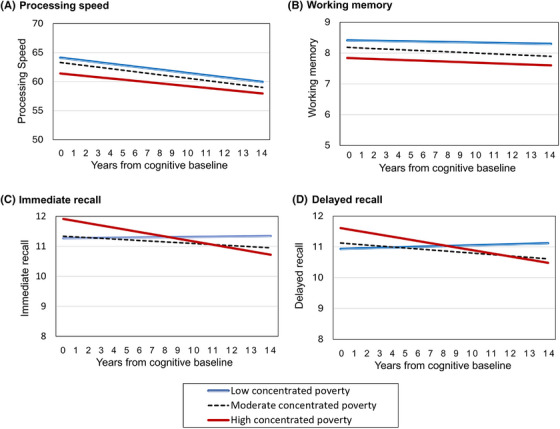
Predicted levels and rates of change in cognitive performance by neighborhood concentrated poverty.

For processing speed (Table 2, Figure 2A), women living in low concentrated poverty showed significantly higher (i.e., better) levels in processing speed between compared to those living in high concentrated poverty areas (estimate = 2.718, 95% CI = [0.104, 5.332]). Although processing speed significantly declined over time, the rate of decline was similar across all concentrated poverty levels. Extrapolated across 10‐year increments, the rate of decline was 4.6% for low concentrated poverty areas, 4.9% for moderate poverty areas, and 4.0% for high concentrated poverty areas relative to baseline.

For working memory (Table 3, Figure 2B), women living in low concentrated poverty areas showed significantly higher levels of working memory compared to those living in high concentrated areas (estimate = 0.574, 95% CI = [0.070, 1.079]). Moderate poverty areas showed small but significant declines in working memory over time, whereas neither low nor high concentrated poverty areas showed significant declines; and there were no statistically significant differences in rates of decline among neighborhood poverty groups.

For immediate recall (Table 4, Figure 2C), women living in low (vs. high) and in moderate (vs. high) concentrated poverty areas showed significantly lower levels of immediate recall (estimate = −0.646, 95% CI = [−1.171, −0.120] for low vs. high; estimate = −0.576, 95% CI = [−1.044, −0.109] for moderate vs. high). Immediate recall showed significant decline for high and moderate concentrated poverty areas; the 10‐year reduction from the baseline score was 7.2% high concentrated poverty areas and 2.4% for moderate poverty areas. The differences between moderate and low concentrated poverty areas (estimate = −0.033, 95% CI = [−0.063, −0.003]), between high and low (estimate = −0.091, 95% CI = [−0.149, −0.032]), and between high and moderate concentrated poverty areas (estimate = −0.058, 95% CI = [−0.112, −0.003]) in rates of immediate recall decline were significant.

For delayed recall (Table 5, Figure 2D), women living in low (vs. high) and in moderate (vs. high) concentrated poverty areas showed significantly lower levels of delayed recall (estimate = −0.674, 95% CI = [−1.194, −0.155] for low vs. high; estimate = −0.487, 95% CI = [−0.947, −0.027] for moderate vs. high). Delayed recall showed significant decline for high and moderate concentrated poverty areas; the 10‐year reduction from the baseline score was 6.9% high concentrated poverty areas and 3.3% for moderate poverty areas. The differences between moderate and low concentrated poverty areas (estimate = −0.050, 95% CI = [−0.079, −0.020]) and between high and low concentrated poverty areas (estimate = −0.094, 95% CI = [−0.151, −0.037]) in rates of delayed recall decline were significant. The pattern of results was maintained when continuous scores of neighborhood concentrated poverty were used (Table S1).

Second, we examined whether the effects of neighborhood concentrated poverty differed across racial groups by including interaction terms of neighborhood × race and neighborhood × time × race. As the interaction effects were significant, we further conducted race‐stratified analysis (Table S2). The pattern of effects of concentrated poverty on decline in episodic memory was mostly maintained except for Japanese women as none of them were living in high concentrated poverty areas. Episodic memory (both immediate and delayed recall) showed significant decline among White, Black, and Chinese women living in high or moderate concentrated poverty areas. The magnitude of effects of high concentrated neighborhood was most pronounced among Black women for episodic memory delayed recall (estimate = −0.107, 95% CI = [−0.188, −0.027]; 10‐year reduction of 9.5% from baseline). The significant pattern of results was maintained for White and Black women when continuous scores of neighborhood concentrated poverty was used. A 1‐SD increase in continuous concentrate poverty score (i.e., better nSES) was associated with slower rates of decline in immediate (estimate = −0.032, 95% CI = [−0.050, −0.014] for Black) and delayed recall (estimate = −0.016, 95% CI = [−0.029, −0.004] for White; estimate = −0.029, 95% CI = [−0.046, −0.012] for Black) over time.

We conducted two sensitivity analyses. First, we used Gi^*^ scores with a second‐order queen contiguity spatial weight matrix, that goes beyond just direct neighborhoods and includes neighboring areas one step away in addition to those that are directly adjacent. The patterns of results did not change, suggesting effects of wide geographic clustering of economic segregation. Second, to examine whether physical activity and cardiovascular disease risk factor would account for the neighborhood concentrated poverty−cognition relationships, we included physical activity and ASCVD as time‐invariant covariates in the above models. After controlling for physical activity and ASCVD, there were small changes in the rates of immediate and delayed recall associated with neighborhood concentrated poverty. However, there were no significant associations of neighborhood concentrated poverty with either PA or ASCVD after controlling for other covariates, indicating limited evidence of mediation.^45^

## 4 DISCUSSION

In this racially and geographically diverse cohort of midlife women, we observed that exposure to greater neighborhood concentrated poverty during midlife was associated with lower initial levels of processing speed and working memory, and with more rapid decline in episodic memory over an average of 10 years throughout midlife. Given that decline in episodic memory during midlife may be indicative of pathologic aging,^46^, ^47^ this result suggests that the risks of developing dementia may begin to emerge as early as midlife period among women living in greater concentrated poverty areas.

Results from our work show that all three groups (i.e., high, moderate, low poverty concentration) showed similar rates of decline in processing speed although there were differences at baseline levels. As processing speed tends to decline earlier in the aging process compared to other cognitive domains,^48^ the results indicate that the magnitude of normative age‐related changes were similar among midlife women living in different levels of concentrated poverty areas. For working memory, neighborhood concentrated poverty was associated with lower initial levels of performance; but differences in rates of decline of working memory among concentrated poverty groups were none or minimal. For episodic memory, however, women living in high concentrated poverty areas experienced a significant reduction in episodic memory over time equivalent to a 10‐year reduction of 7.2% (for immediate recall) and 6.9% (for delayed recall) from baseline respectively. Given that episodic memory typically remains stable or shows only small age‐related decline through midlife,^49^, ^50^ a notable decline in episodic memory during midlife period may signal potential pathologic aging,^46^, ^47^ suggesting that neighborhood concentrated poverty could be an important factor contributing to the risk of ADRD beyond normative cognitive aging processes.

Although our findings are largely consistent with prior studies^8^, ^11^, ^12^, ^20^ that demonstrated the protective role of greater nSES on individuals’ cognitive health, only a few studies have examined long‐term changes in specific cognitive domains.^8^, ^20^ Hunt and colleagues found significant effects of nSES on long‐term changes in executive function/processing speed, but not in memory/learning.^8^ In contrast, Rosso and colleagues did not find a significant association between nSES and processing speed over 6 years.^20^ It is not clear why the results are conflicting. Different definition of nSES, different sample demographics (e.g., sex, age, race), the geographic locations of participants, the durations of follow‐up, or the assessment timing may explain the discrepancies. In addition, we found that neither physical activity nor cardiovascular risk accounted for the effects of neighborhood concentrated poverty on episodic memory decline, although a more adverse cardiovascular risk factor profile was associated with faster rates of decline in processing speed.^31^ Different types of risk factors (e.g., contextual‐level nSES, individual‐level physical activity, cardiovascular disease risk factors) appeared to be related to cognitive function through potentially different mechanisms (e.g., vascular pathway, AD pathology, cognitive reserve).^8^, ^20^, ^31^, ^51^, ^52^ Given that midlife is a critical period when adverse cognitive changes in processing speed and verbal episodic memory become evident,^30^ it is important to understand underlying pathways through which different risk factors are associated with different cognitive domains to reveal points of possible interventions.

We further examined whether the effects of concentrated poverty on cognition differed across racial groups. Black women living in high concentrated poverty areas experienced the most rapid decline in episodic memory delayed recall over time; the 10‐year reduction from the baseline score was 9.5% for Black women compared to 4.1% for White women. It is possible that racial and ethnic minorities especially Black individuals face combined disadvantages from both individual (e.g., racial discrimination) and environmental (e.g., nSES) factors,^53^, ^54^ which may lead to greater cognitive impairment. But caution is required in interpretation of these findings due to the small sample sizes in racial/ethnic groups, different geographic locations by race, and different racial distribution by neighborhood poverty. Meyer and colleagues examined four combinations of racial segregation and nSES (i.e., high/low racial segregation × high/low nSES) on rates of cognitive change over time.^55^ They found that distinct combinations of neighborhood characteristics were linked to decline in episodic memory in nuanced ways. For example, Black participants seemed to benefit from living in neighborhoods with higher clustering of Black residents if nSES was high, or from living in areas with low clustering of Black residents if nSES was low. The small sample sizes of each group, ranging from *N* = 75 to 88, however, limit generalizability. Given limited evidence, future studies are required to investigate the complex interplay between racial and economic segregation on cognitive changes.

This study went beyond the previous literature by examining effects of the geospatial distribution of adjacent neighborhood deprivation, rather than focusing on neighborhood deprivation within a single census tract or at single point in time, on changes in various cognitive domains. People are likely to travel across multiple neighborhoods throughout the day, and individuals’ community context is likely to include nearby areas outside of a given neighborhood.^23^, ^56^, ^57^ Therefore, individuals’ health‐related behaviors and health outcomes may be impacted by both immediate and surrounding neighborhoods.^57^, ^58^ For example, disadvantaged neighborhoods are likely to have limited access/opportunities for the high quality built environment features (e.g., recreation centers, art centers and museums, libraries) that can provide cognitive enrichment.^6^ In addition, poor access to health care facilities in deprived areas may increase the likelihood of cognitive impairment possibly through failure to manage other health conditions and diseases.^59^ People living in concentrated poverty areas are also likely to be exposed to more neurotoxic air pollution, which can accelerate neurodegenerative processes through cerebrovascular disease and Aβ deposition.^60^, ^61^ Concentrated poverty areas have high rates of violent crime,^62^ which leads to lower perceived safety and activate heightened emotional and physiological stress responses, and eventually impair brain and cognitive health.^63^, ^64^, ^65^ Because people's social networks are also in part geographically bounded, those living in high poverty areas are likely to have less social capital to link them to public and private resources related to health‐related behaviors and health outcomes.^66^

Importantly, clustering of deprived neighborhoods can make the above‐mentioned disadvantages worse and prevent residents living in high concentrated poverty areas from accessing better resources in affluent neighborhoods. Prior research indicated that, although the average travel distances among people living in the metropolitan area were similar across neighborhood income characteristics, residents of high and low nSES tend to carry out their daily routine activities (e.g., working, shopping, worshipping, seeing a doctor) in mostly nonoverlapping parts of the city.^57^, ^67^ Thus, segregation not only occurs in places where people live but also where they travel throughout the city and impacts to whom and what they are exposed.^57^ If residents of deprived neighborhood mostly travel to neighboring, similarly deprived neighborhoods, it will reinforce spatial segregation and inequality for cognitive health. As we could not examine this exact mechanism in the current study, future research is required to confirm this spatial segregation pattern using real‐time GPS technologies.

There are some limitations to this study. First, our measure of neighborhood concentrated poverty only used poverty data and did not take other nSES factors into account. As accumulating evidence suggests the importance of the composite nSES variable (e.g., neighborhood income, education, housing) on cognitive outcomes,^7^, ^10^, ^11^, ^22^ future studies would need to include multiple nSES factors for spatial clustering. Second, each site was highly variable in demographics (e.g., racial composition) as well as physical and social neighborhood characteristics (e.g., population density), and participants may not have been representative of the overall populations in each city. Thus, our work lacks generalizability within each site or in rural settings. Third, although we measured neighborhood concentrated poverty frequently over midlife, we could not account for the effects of early life neighborhood features on cognition. Fourth, we focused on residential neighborhoods around participants’ home address, but we could not account for work neighborhoods or other places that participants regularly visit. Fifth, future studies need to identify which sub‐populations or individual characteristics (e.g., individual‐level education, income^22^, ^68^, ^69^) render an individual more or less vulnerable to the effects of neighborhood concentrated poverty. Identifying subpopulations vulnerable to nSES would be able to inform targeted and effective intervention and prevention strategies. Sixth, this study was unable to examine whether segregation in day‐to‐day travel patterns contributes to the current findings with neighborhood concentrated poverty. Future research should investigate the underlying behavioral mechanisms using real‐time GPS technologies.

## 5 CONCLUSION

We found that living in areas with high concentrated poverty was associated with faster rates of decline in episodic memory among women over an average of 10 years of midlife. This pattern was most pronounced among Black women. However, we did not find any significant differences in rates of decline in processing speed or working memory by levels of concentrated poverty.

Given that episodic memory impairment is a key characteristic of the early stages of ADRD, decline in episodic memory during midlife may indicate risk for future cognitive impairment.^46^, ^47^, ^49^, ^50^ Thus, midlife women living in greater neighborhood concentrated poverty areas may require timely interventions to ameliorate cognitive health disparities. More investigation is needed to identify the specific social and biological mechanisms underlying this association and ultimately to explore policy and environmental interventions that mitigate cognitive health disparities in economically segregated areas.

## CONFLICT OF INTEREST STATEMENT

The authors declare no conflicts of interest. Author disclosures are available in the Supporting Information.

## CONSENT STATEMENT

All participants provided written informed consent at each study visit, and approval was obtained from Institutional Review Boards at each SWAN clinical site.

## Supporting information



Supporting Information

Supporting Information
