# Supplementary material for: Exposure to neighborhood concentrated poverty is associated with faster decline in episodic memory among midlife women
Source: Alzheimers Dement. 2025 Apr 6;21(4):e70139. doi: 10.1002/alz.70139 (PMC11972984; doi:10.1002/alz.70139)
Supplement: Supplementary file 1 — Supporting Information [file ALZ-21-e70139-s002.docx]

# SUPPLEMENTARY MATERIALS

Supplementary Figure 1. Flowchart for analysis sample.


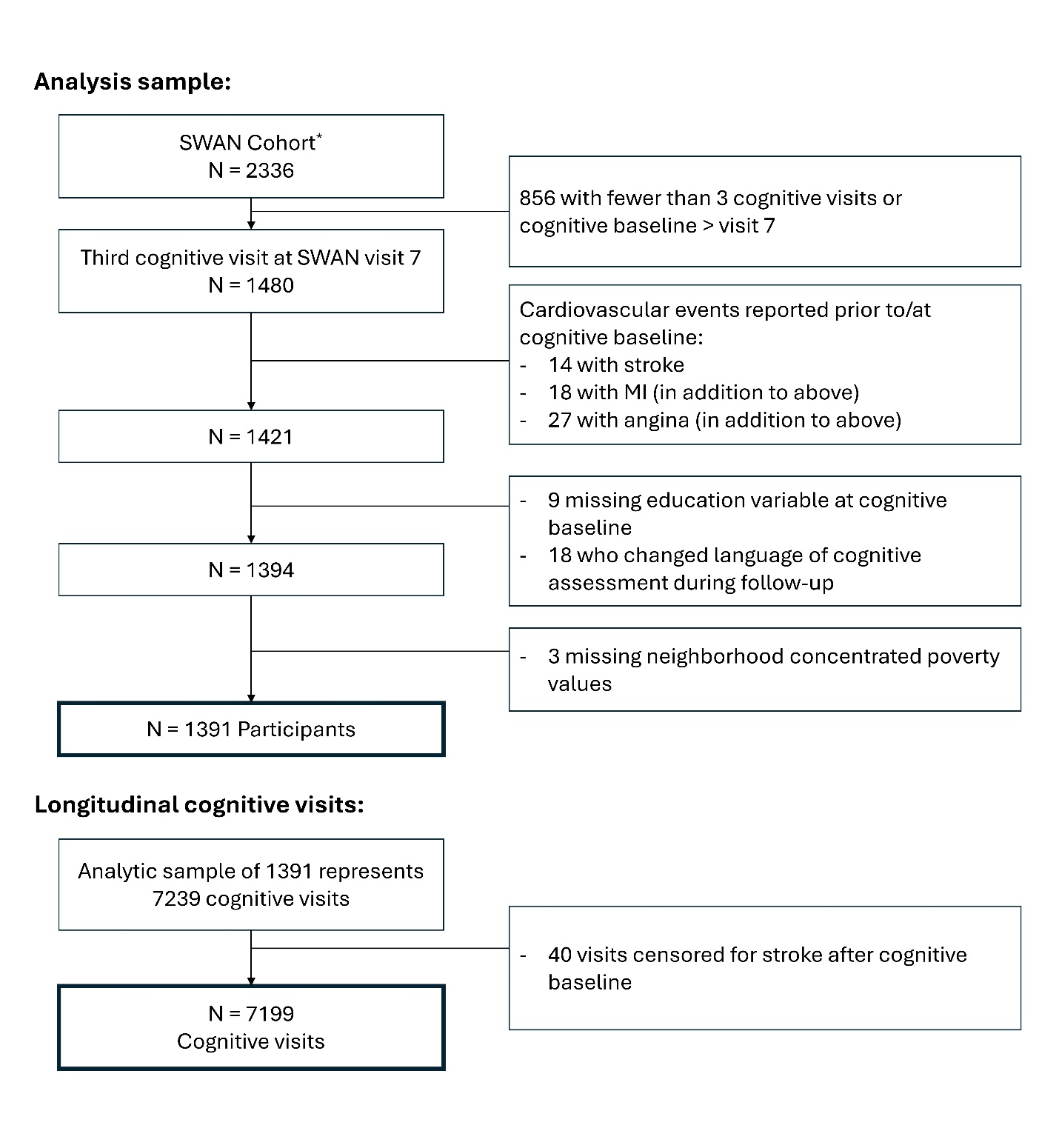


*Note.* MI: myocardial infarction; SWAN: Study of Women’s Health Across the Nation.
* Initial sample included participants from five U.S. study sites: Chicago, IL; Detroit, MI; Pittsburgh, PA; Oakland, CA; and Los Angeles, CA.

Supplementary Table 1. Estimated levels and changes in cognition by race.

| Cognitive domain | Neighborhood concentrated poverty | Estimate | SE | 95% CI | |
| --- | --- | --- | --- | --- | --- |
|  |  |  |  | Lower | Upper |
| Processing speed | nSES | -0.360 | 0.195 | -0.742 | 0.021 |
|  | nSES × Time | -0.010 | 0.010 | -0.029 | 0.010 |
| Working memory | nSES | -0.046 | 0.040 | -0.124 | 0.033 |
|  | nSES × Time | -0.002 | 0.003 | -0.007 | 0.003 |
| Immediate recall | nSES | 0.069 | 0.039 | -0.009 | 0.146 |
|  | nSES × Time | -0.012 | 0.004 | -0.020 | -0.004 |
| Delayed recall | nSES | 0.095 | 0.039 | 0.018 | 0.172 |
|  | nSES × Time | -0.016 | 0.004 | -0.024 | -0.008 |

*Note*. nSES indicates *continuous* score of neighborhood concentrated poverty, with higher score representing less concentrated neighborhood poverty (i.e., better nSES). A 1-unit change in nSES refers to a 1-SD change. Covariates were baseline age, education, study site (time-invariant), pay for basics, smoking, alcohol use, menopausal status, and hormone use (time-varying).

Supplementary Table 2. Estimated levels and changes in cognition by race.

1a. White (N=679)

| Cognitive domain | Neighborhood concentrated poverty | Estimate | SE | 95% CI | |
| --- | --- | --- | --- | --- | --- |
|  |  |  |  | Lower | Upper |
| Processing speed | Low | 64.593 | 0.933 | 62.763 | 66.423 |
|  | Moderate | 63.445 | 0.815 | 61.846 | 65.043 |
|  | High | 62.687 | 2.320 | 58.136 | 67.238 |
|  | Low × Time | -0.324 | 0.043 | -0.408 | -0.240 |
|  | Moderate × Time | -0.336 | 0.031 | -0.398 | -0.274 |
|  | High × Time | -0.234 | 0.093 | -0.416 | -0.051 |
| Working memory | Low | 8.539 | 0.244 | 8.061 | 9.016 |
|  | Moderate | 8.330 | 0.221 | 7.896 | 8.764 |
|  | High | 7.712 | 0.416 | 6.895 | 8.529 |
|  | Low × Time | -0.025 | 0.013 | -0.052 | 0.001 |
|  | Moderate × Time | -0.020 | 0.008 | -0.037 | -0.004 |
|  | High × Time | -0.004 | 0.027 | -0.057 | 0.050 |
| Immediate recall | Low | 11.267 | 0.212 | 10.851 | 11.682 |
|  | Moderate | 11.110 | 0.287 | 10.548 | 11.672 |
|  | High | 11.305 | 0.482 | 10.361 | 12.250 |
|  | Low × Time | 0.022 | 0.019 | -0.016 | 0.060 |
|  | Moderate × Time | -0.048 | 0.013 | -0.074 | -0.023 |
|  | High × Time | -0.049 | 0.049 | -0.144 | 0.047 |
| Delayed recall | Low | 10.894 | 0.206 | 10.489 | 11.299 |
|  | Moderate | 10.955 | 0.278 | 10.411 | 11.500 |
|  | High | 11.338 | 0.466 | 10.424 | 12.251 |
|  | Low × Time | 0.034 | 0.018 | -0.002 | 0.069 |
|  | Moderate × Time | -0.047 | 0.012 | -0.072 | -0.023 |
|  | High × Time | -0.046 | 0.046 | -0.137 | 0.044 |

*Note*. Covariates were baseline age, education, study site (time-invariant), pay for basics, smoking, alcohol use, menopausal status, and hormone use (time-varying).

1b. Black (N=330)

| Cognitive domain | Neighborhood concentrated poverty | Estimate | SE | 95% CI | |
| --- | --- | --- | --- | --- | --- |
|  |  |  |  | Lower | Upper |
| Processing speed | Low | 56.493 | 1.438 | 53.671 | 59.315 |
|  | Moderate | 57.126 | 1.053 | 55.060 | 59.192 |
|  | High | 57.924 | 1.912 | 54.172 | 61.676 |
|  | Low × Time | -0.282 | 0.147 | -0.570 | 0.006 |
|  | Moderate × Time | -0.334 | 0.043 | -0.419 | -0.249 |
|  | High × Time | -0.337 | 0.111 | -0.555 | -0.119 |
| Working memory | Low | 6.957 | 0.726 | 5.531 | 8.382 |
|  | Moderate | 6.909 | 0.250 | 6.419 | 7.399 |
|  | High | 6.563 | 0.394 | 5.789 | 7.336 |
|  | Low × Time | 0.002 | 0.062 | -0.119 | 0.123 |
|  | Moderate × Time | -0.032 | 0.011 | -0.054 | -0.011 |
|  | High × Time | -0.020 | 0.020 | -0.060 | 0.019 |
| Immediate recall | Low | 9.665 | 0.554 | 8.579 | 10.752 |
|  | Moderate | 10.525 | 0.791 | 8.975 | 12.075 |
|  | High | 11.817 | 0.893 | 10.068 | 13.567 |
|  | Low × Time | 0.121 | 0.072 | -0.020 | 0.261 |
|  | Moderate × Time | 0.014 | 0.017 | -0.019 | 0.047 |
|  | High × Time | -0.090 | 0.044 | -0.175 | -0.004 |
| Delayed recall | Low | 9.599 | 0.576 | 8.470 | 10.727 |
|  | Moderate | 10.304 | 0.822 | 8.693 | 11.915 |
|  | High | 11.269 | 0.922 | 9.463 | 13.076 |
|  | Low × Time | 0.069 | 0.068 | -0.064 | 0.203 |
|  | Moderate × Time | -0.009 | 0.016 | -0.041 | 0.023 |
|  | High × Time | -0.107 | 0.041 | -0.188 | -0.027 |

*Note*. Covariates were baseline age, education, study site (time-invariant), pay for basics, smoking, alcohol use, menopausal status, and hormone use (time-varying).

1c. Chinese (N=168)

| Cognitive domain | Neighborhood concentrated poverty | Estimate | SE | 95% CI | |
| --- | --- | --- | --- | --- | --- |
|  |  |  |  | Lower | Upper |
| Processing speed | Low | 66.537 | 2.519 | 61.589 | 71.484 |
|  | Moderate | 61.832 | 0.866 | 60.130 | 63.534 |
|  | High | 57.349 | 2.297 | 52.836 | 61.862 |
|  | Low × Time | -0.451 | 0.187 | -0.817 | -0.084 |
|  | Moderate × Time | -0.255 | 0.056 | -0.366 | -0.144 |
|  | High × Time | -0.044 | 0.109 | -0.258 | 0.171 |
| Working memory | Low | 7.565 | 0.543 | 6.498 | 8.631 |
|  | Moderate | 7.024 | 0.194 | 6.643 | 7.404 |
|  | High | 6.474 | 0.535 | 5.423 | 7.525 |
|  | Low × Time | 0.059 | 0.039 | -0.018 | 0.136 |
|  | Moderate × Time | -0.029 | 0.016 | -0.059 | 0.002 |
|  | High × Time | -0.036 | 0.042 | -0.119 | 0.046 |
| Immediate recall | Low | 10.390 | 0.415 | 9.577 | 11.204 |
|  | Moderate | 10.770 | 0.590 | 9.613 | 11.927 |
|  | High | 11.284 | 0.703 | 9.905 | 12.662 |
|  | Low × Time | -0.045 | 0.047 | -0.136 | 0.047 |
|  | Moderate × Time | -0.053 | 0.016 | -0.085 | -0.021 |
|  | High × Time | -0.109 | 0.046 | -0.199 | -0.019 |
| Delayed recall | Low | 10.396 | 0.416 | 9.582 | 11.211 |
|  | Moderate | 10.844 | 0.591 | 9.686 | 12.002 |
|  | High | 10.858 | 0.701 | 9.483 | 12.232 |
|  | Low × Time | -0.069 | 0.048 | -0.163 | 0.024 |
|  | Moderate × Time | -0.089 | 0.017 | -0.122 | -0.056 |
|  | High × Time | -0.065 | 0.047 | -0.157 | 0.026 |

*Note*. Covariates were baseline age, education, study site (time-invariant), pay for basics, smoking, alcohol use, menopausal status, and hormone use (time-varying).

1d. Japanese (N=214)

| Cognitive domain | Neighborhood concentrated poverty | Estimate | SE | 95% CI | |
| --- | --- | --- | --- | --- | --- |
|  |  |  |  | Lower | Upper |
| Processing speed | Low | 65.190 | 0.992 | 63.243 | 67.138 |
|  | Moderate | 65.014 | 0.778 | 63.487 | 66.542 |
|  | Low × Time | -0.244 | 0.045 | -0.332 | -0.157 |
|  | Moderate × Time | -0.216 | 0.045 | -0.305 | -0.127 |
| Working memory | Low | 6.801 | 0.216 | 6.377 | 7.226 |
|  | Moderate | 6.390 | 0.207 | 5.985 | 6.796 |
|  | Low × Time | 0.022 | 0.017 | -0.011 | 0.055 |
|  | Moderate × Time | 0.014 | 0.015 | -0.014 | 0.043 |
| Immediate recall | Low | 11.319 | 0.218 | 10.890 | 11.747 |
|  | Moderate | 11.265 | 0.322 | 10.633 | 11.896 |
|  | Low × Time | -0.051 | 0.022 | -0.094 | -0.009 |
|  | Moderate × Time | -0.001 | 0.020 | -0.040 | 0.037 |
| Delayed recall | Low | 11.056 | 0.223 | 10.620 | 11.493 |
|  | Moderate | 10.939 | 0.328 | 10.296 | 11.581 |
|  | Low × Time | -0.029 | 0.024 | -0.077 | 0.019 |
|  | Moderate × Time | 0.018 | 0.022 | -0.025 | 0.061 |

*Note*. Covariates were baseline age, education, study site (time-invariant), pay for basics, smoking, alcohol use, menopausal status, and hormone use (time-varying).
